# Towards malaria elimination in Savannakhet, Lao PDR: mathematical modelling driven strategy design

**DOI:** 10.1186/s12936-017-2130-3

**Published:** 2017-11-28

**Authors:** Sai Thein Than Tun, Lorenz von Seidlein, Tiengkham Pongvongsa, Mayfong Mayxay, Sompob Saralamba, Shwe Sin Kyaw, Phetsavanh Chanthavilay, Olivier Celhay, Tran Dang Nguyen, Thu Nguyen-Anh Tran, Daniel M. Parker, Maciej F. Boni, Arjen M. Dondorp, Lisa J. White

**Affiliations:** 1 Mahidol Oxford Tropical Medicine Research Unit, Faculty of Tropical Medicine, Mahidol University, Bangkok, Thailand; 2 Centre for Tropical Medicine and Global Health, Nuffield Department of Medicine, Churchill Hospital, Oxford, UK; 3 Savannakhet Provincial Health Department, Phonsavangnuea Village, Kaysone-Phomvihan District, Savannakhet, Lao People’s Democratic Republic; 4 Lao-Oxford-Mahosot Hospital-Wellcome Trust Research Unit (LOMWRU), Microbiology Laboratory, Vientiane, Lao People’s Democratic Republic; 5 Faculty of Postgraduate Studies, University of Health Sciences, Vientiane, Lao People’s Democratic Republic; 6 Institute of Francophonie for Tropical Medicine, Vientiane, Lao People’s Democratic Republic; 7 Oxford University Clinical Research Unit, Wellcome Trust Major Overseas Programme, Ho Chi Minh City, Vietnam; 8 Shoklo Malaria Research Unit, Mahidol-Oxford Tropical Medicine Research Unit, Mahidol University, Mae Sot, Thailand; 9 Department of Biology, Pennsylvania State University, University Park, PA USA

**Keywords:** Malaria elimination, *Plasmodium falciparum*, Mathematical model, Savannakhet, Laos, Mass Vaccination and Drug Administration (MVDA), Malaria surveillance

## Abstract

**Background:**

The number of *Plasmodium falciparum* malaria cases around the world has decreased substantially over the last 15 years, but with the spread of resistance against anti-malarial drugs and insecticides, this decline may not continue. There is an urgent need to consider alternative, accelerated strategies to eliminate malaria in countries like Lao PDR, where there are a few remaining endemic areas. A deterministic compartmental modelling tool was used to develop an integrated strategy for *P. falciparum* elimination in the Savannakhet province of Lao PDR. The model was designed to include key aspects of malaria transmission and integrated control measures, along with a user-friendly interface.

**Results:**

Universal coverage was the foundation of the integrated strategy, which took the form of the deployment of community health workers who provided universal access to early diagnosis, treatment and long-lasting insecticidal nets. Acceleration was included as the deployment of three monthly rounds of mass drug administration targeted towards high prevalence villages, with the addition of three monthly doses of the RTS,S vaccine delivered *en masse* to the same high prevalence sub-population. A booster dose of vaccine was added 1 year later. The surveillance-as-intervention component of the package involved the screening and treatment of individuals entering the simulated population.

**Conclusions:**

In this modelling approach, the sequential introduction of a series of five available interventions in an integrated strategy was predicted to be sufficient to stop malaria transmission within a 3-year period. These interventions comprised universal access to early diagnosis and adequate treatment, improved access to long-lasting insecticidal nets, three monthly rounds of mass drug administration together with RTS,S vaccination followed by a booster dose of vaccine, and screening and treatment of imported cases.

**Electronic supplementary material:**

The online version of this article (10.1186/s12936-017-2130-3) contains supplementary material, which is available to authorized users.

## Background

As has been seen in many areas around the world, the number of malaria cases reported in the Lao People’s Democratic Republic (Lao PDR) has significantly decreased over the last decade [1]. Encouraged by this success, the Laos Ministry of Health aims to eliminate malaria by 2030 [2]. This undertaking is threatened by the recent emergence and spread of *Plasmodium falciparum* strains resistant to first-line anti-malarials, the artemisinin‐based combination therapy (ACT) [3]. The current steady decline in *P. falciparum* transmission may not continue in Lao PDR and other countries in the Greater-Mekong Subregion (GMS). Moreover, should multidrug resistance continue to spread through Asian countries unchecked, its inevitable arrival on the African continent will lead to a public health disaster [4, 5]. In response to this threat, the current World Health Organization (WHO) strategy is to eliminate *P. falciparum* malaria from the GMS with alacrity [6]. The WHO Global Technical Strategy for malaria 2016–2030 includes: (1) ensuring universal access to malaria prevention, diagnosis and treatment; (2) accelerating efforts towards elimination and attainment of malaria-free status; (3) transforming malaria surveillance into a core intervention.

The current coverage of early detection and treatment, along with the distribution of long-lasting insecticide-treated bed nets (LLIN), may be insufficient to achieve this strategy. There is an urgent need to consider additional alternative strategies to accelerate the elimination of malaria in countries like Lao PDR, and while ACT is still generally effective [7].

One such strategy is the elimination of the entire parasite reservoir by the presumptive treatment of all residents in malaria-endemic areas. Mass drug administrations (MDA) have successfully eliminated malaria on a Pacific island [8], but require the participation of the majority of the community. MDA provide only temporary (4–6 months) reductions in malaria cases if coverage is less than 80% or if infected people re-import malaria into the area [9, 10]. To interrupt transmission permanently, additional interventions would probably be needed, such as the inclusion of mass vaccination using RTS,S/AS01 given in three doses at monthly intervals [1]. This vaccine does not provide life-long, complete protection but it is efficacious for several months. Protection wanes to around 50% at 200 days after the third dose [11]. A combined Mass Vaccination and Drug Administration (MVDA) clears the parasite reservoir in a population and protects participants for a limited period against infections. This parasite-free window provides sufficient time to clear on-going sources of parasite transmission such as new arrivals in the community, and allows for the rolling out of the intervention to surrounding villages, thus eliminating future sources of malaria transmission.

It is now widely accepted that the elimination of malaria will require several stages of programmatic scale-up [12]. Current and novel tools and their effective use have been highlighted as essential to achieve malaria eradication. For example, the Bill and Melinda Gates Foundation has urged the malaria community to consider the following three aims [13]:

- Complete detection: health workers need to find all individuals who have the parasite in their blood, regardless of whether they are symptomatic or not.
- Complete cure: using treatments that clear all malaria parasites.
- Complete prevention: reducing opportunities for mosquitoes to transmit parasites to the human host.

Each of these aims will require new products to fully meet the challenge. For relatively low transmission settings like those in the GMS, and given the urgency of the drug resistance issue in this region, there is a compelling argument that these aims could be met with existing tools which provide:

- Partial detection in the form of village health volunteers (VHV) screening new arrivals with high sensitivity rapid diagnostic tests (HS RDT).
- Partial cure with ACT treating most clinical infections but with some treatment failure, and focal MDA to treat most infections at three time points.
- Partial prevention, with LLIN offering a reduction of 30% in the force of infection [14], and RTS,S mass vaccination offering temporary and partial protection against infection.

The sequential inclusion of each component to build an integrated strategy was done because, with the exception of MDA and RTS,S, these interventions are routine in Lao PDR. MDA is, however, being trialled [15]. The addition of RTS,S is also operationally highly feasible since it can be delivered through the same mechanism.

Here, a simple mathematical model was used to evaluate whether a suite of detection and treatment approaches for malaria elimination can eliminate malaria in one specific geographically defined area, namely Savannakhet province, Lao PDR. The model structure used was similar to that of the mathematical model which predicted the current emergence of multi-drug resistance in the GMS [16, 17]. Such model structures have been used as tools to support policy making [16–19] and have repeatedly proven to be both agile and qualitatively accurate for malaria strategy exploration [20, 21]. When exploring combinations of novel interventions in settings where data are sparse, simple models can be applied more readily than their more accurate, but data-hungry, complex counterparts, and are adequate for preliminary simulations to support the design of field trials where more accurate data could be collected. For example, in 2012 a model predicted that three rounds of MDA in 3 months would be an optimal MDA strategy [22], a design that is currently under trial in the GMS [23]. Previous modelling work has indicated that the delivery of vaccination simultaneously during an elimination could act to increase the impact of the intervention, while simultaneously slowing the spread of anti-malarial drug resistance [21]. The addition of vaccination is, therefore, included in the elimination package explored here for Savannakhet.

## Methods

### Study site

**Fig. 1:**
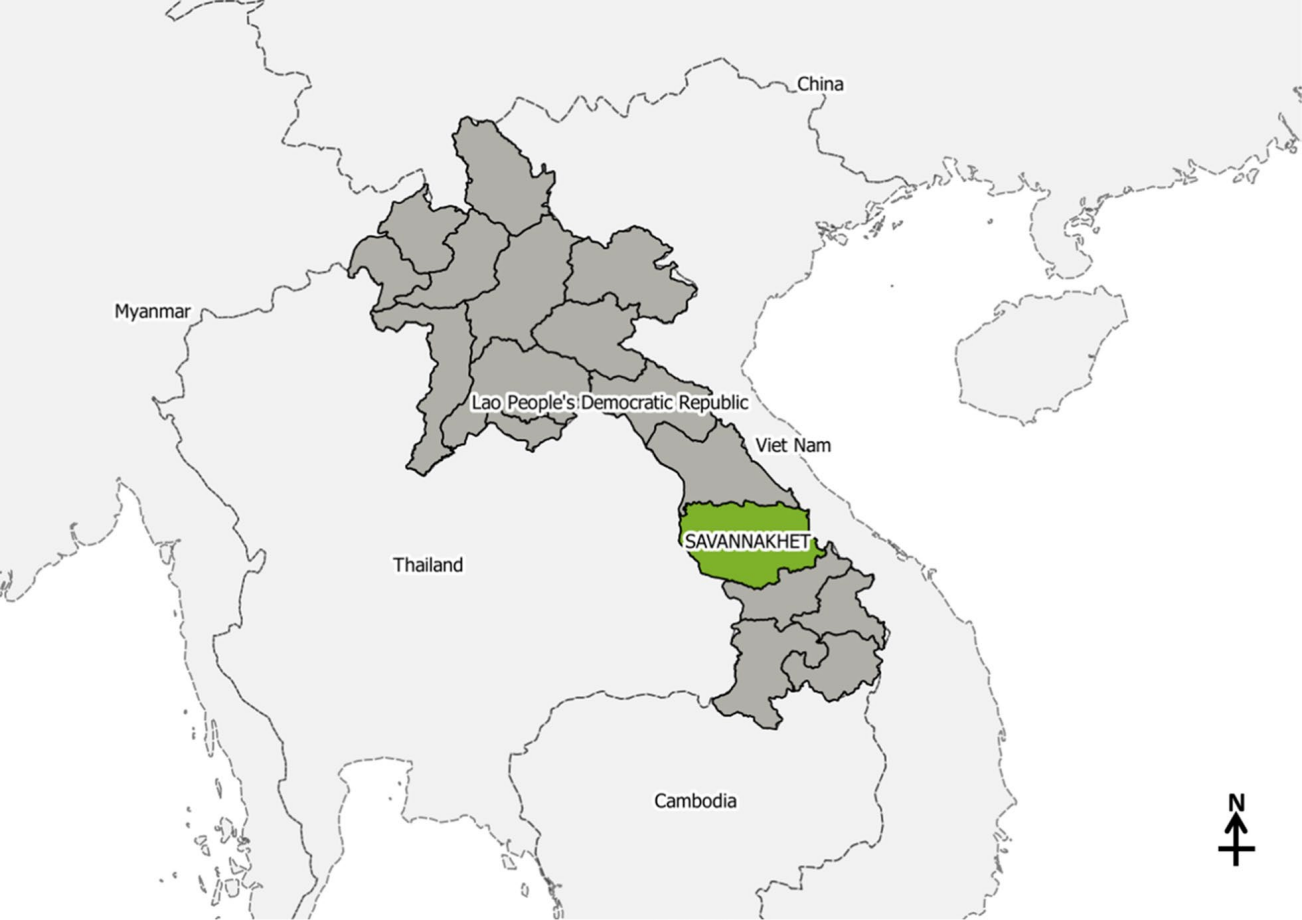
Savannakhet Province in Lao People’s Democratic Republic

**Fig. 2:**
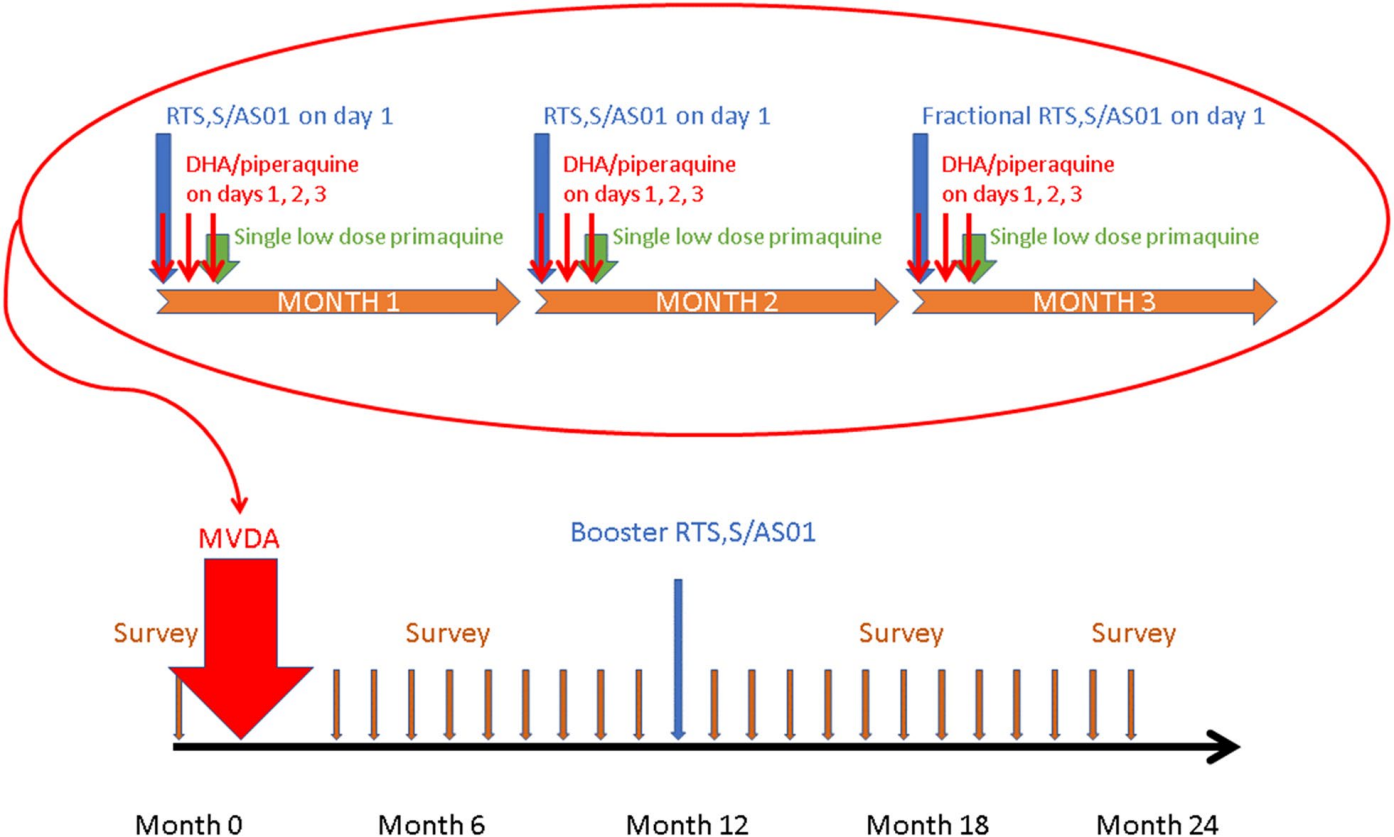
Timeline for Mass Vaccination and Drug Administration (MVDA), booster administration, and follow-up

Savannakhet (Fig. 1), the most populated province in Lao PDR [24], was used to simulate the impact of combined interventions (Fig. 2). Among the 18 provinces of Lao PDR, Savannakhet has the third highest malaria incidence [25]. A survey conducted in 18 rural villages in Savannakhet in 2015, using an ultrasensitive quantitative PCR approach (uPCR), detected asymptomatic *Plasmodium* infections in 175 out of 888 samples (20%), of which 3.6% were *P. falciparum* [25]. Most villages in Savannakhet are relatively accessible and malaria elimination is a high priority for the local government. Starting in April 2016, a pilot project to eliminate malaria was initiated in four villages in the Nong district of Savannakhet. The project showed that MDA in Savannakhet is both feasible and well accepted; more than 80% of targeted villagers participated in the three rounds of drug administration [26].

### The interventions

Early diagnosis and adequate treatment (EDAT) involves comprehensive case management that includes the use of appropriate standard rapid diagnostic tests (RDT) to detect symptomatic *P. falciparum* and *Plasmodium vivax* infections and, if available, glucose-6-phosphate dehydrogenase (G6PD) deficiency, plus treatment following government guidelines, which consists of ACT for *P. falciparum* and a schizontocidal drug combined with 14 days of primaquine therapy for *P. vivax*. LLIN remain the only widely recommended and used form of vector control in Lao PDR. The impact of LLIN is limited by the outdoor, daytime biting behaviour of the important local malaria vectors [27, 28]. In the absence of alternative, proven vector control methods, access for every resident to an intact LLIN will be part of the strategy. EDAT and LLIN provision will be implemented by VHV in each village in the study area. Other roles of the VHV include the reporting of diagnosed and treated malaria cases, and the engagement of the wider community in the malaria elimination project. It is assumed that the recruitment, training, and deployment of new VHV will take 1 year.

Communities with a high number of malaria cases (as reported by VHV) will be targeted for MDA and mass vaccination (MVDA). The current MDA regimen consists of three rounds of three doses of dihydroartemisinin/piperaquine (DHA-PPQ, 40/320 mg) and a single low dose of primaquine (0.25 mg/kg irrespective of G6PD status for gametocidal action against *P. falciparum*) with each round. One dose of RTS,S/AS01 will be added to each round of anti-malarial drugs, and there will be a booster dose after 1 year (Fig. 2).

Once all of the *P. falciparum* infections have been cleared, there remains a risk for re-importation of infections. Visitors and immigrants will be asked to provide a finger prick blood sample for rapid testing with a new generation lateral flow assay (HS RDT such as Alere Malaria Ag *P. f.*) which has 44% sensitivity and 99.8% specificity in low-transmission setting when uPCR is taken as Ref. [12]. Any infected newcomers will be offered appropriate treatment for their infection.

### The modelling tool

**Fig. 3:**
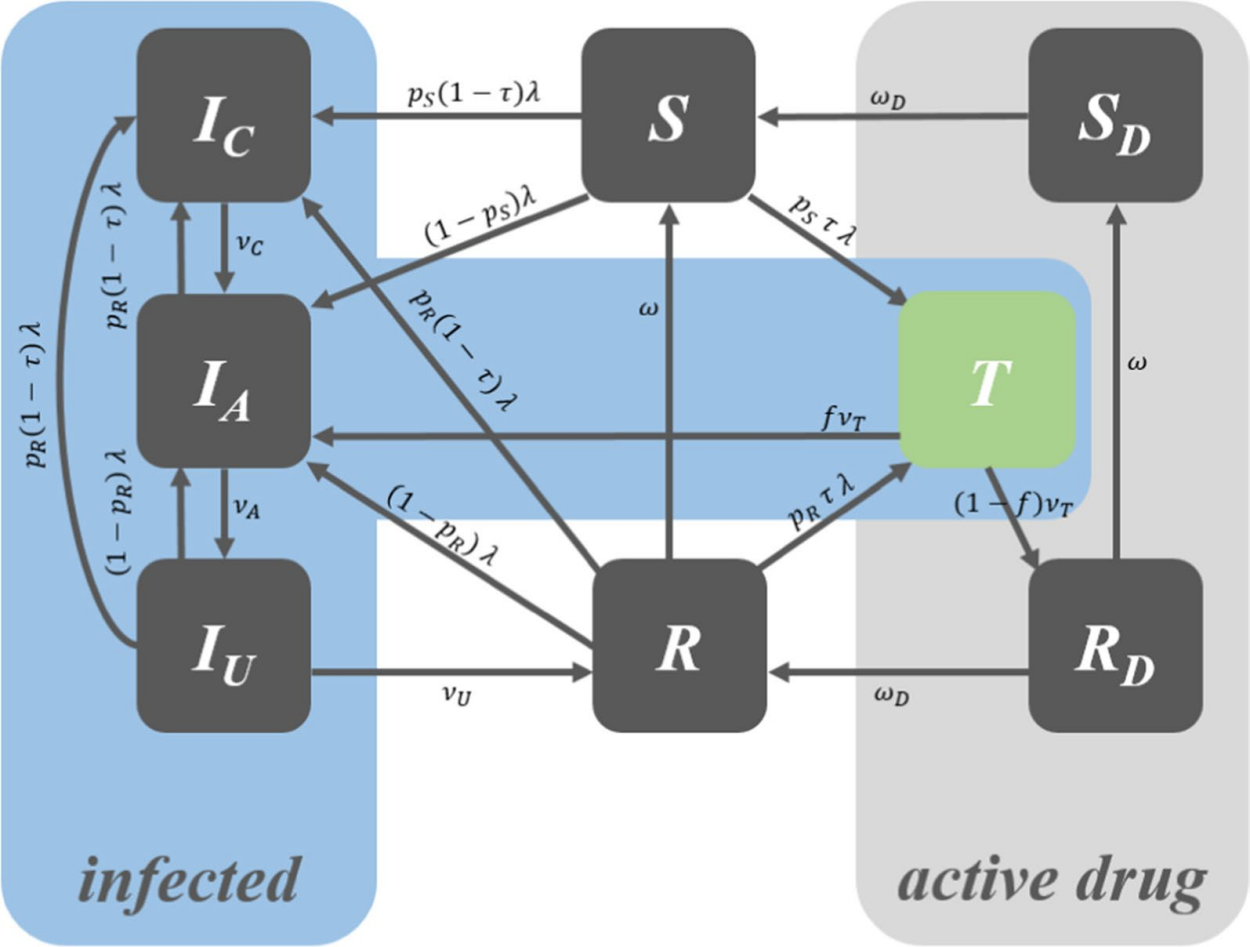
Model structure

**Fig. 4:**
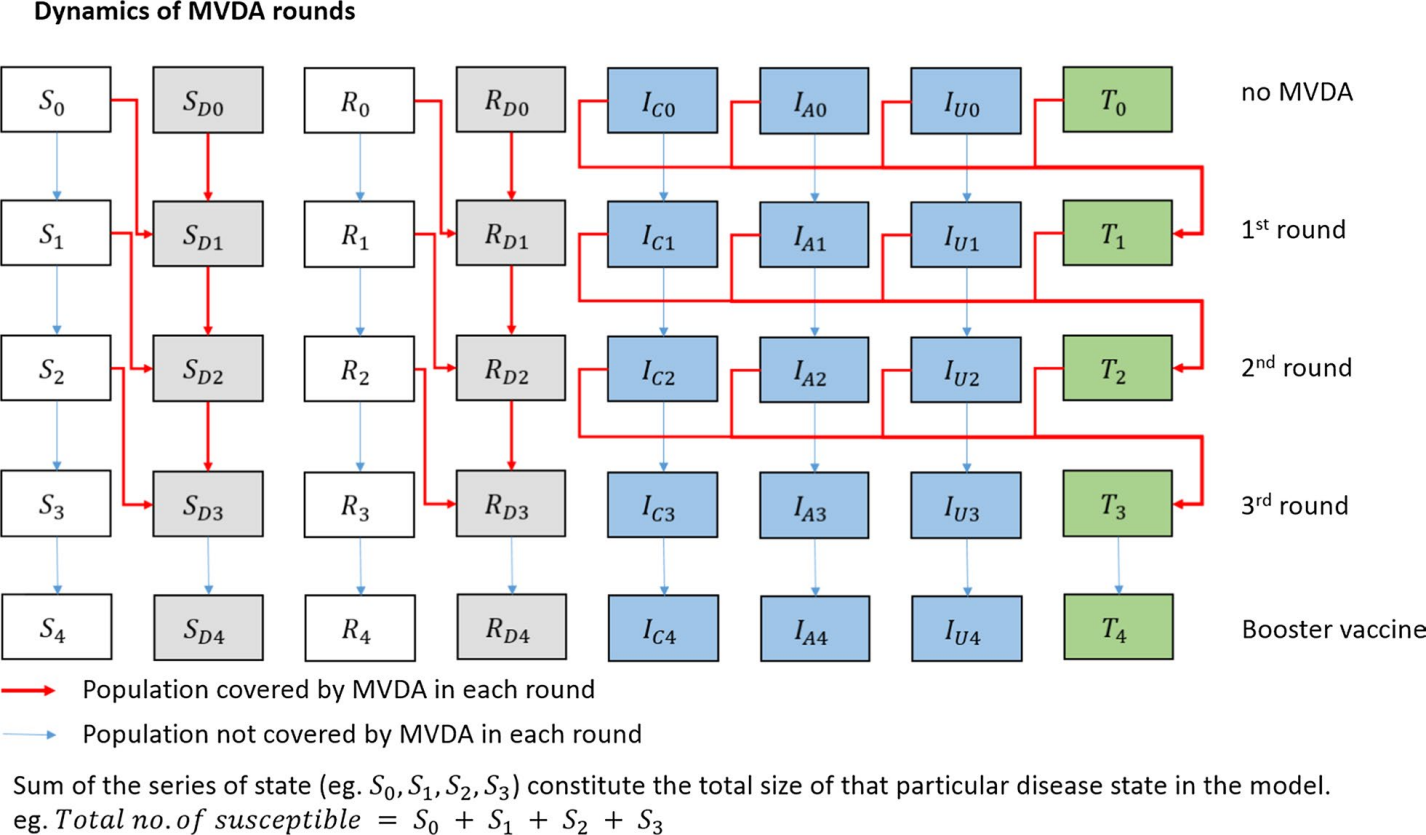
Dynamics of the model

A simple, web-based deterministic compartmental modelling tool, developed using the R programming language and software [29] with shiny [30], deSolve [31], TSA [32] and Rcpp [33] packages, was used for this modelling exercise. The model is an SITR (Susceptible-Infected-Treatment-Recovered) model that includes eight compartments, in which different levels of infected states are included to account for the asymptomatic malaria cases. Cases in the “Infected, Asymptomatic & Patent” (I_A_) compartment will be detectable by microscopy and RDT, whereas cases in “Infected, Asymptomatic & Sub-microscopic” (I_U_) will not be detected with such tests. The model structure for infection dynamics and treatment dynamics is illustrated in Figs. 3 and 4.

The modelling tool can be accessed at [34].

Various baseline and intervention scenarios can be set up using the sliders on the tool. The increase in coverage and the addition of newer interventions are modelled to start after year 2018. Based on any particular set-up, the model will predict the expected monthly incidence of both detected and total clinical *P. falciparum* malaria cases per 1000 population per month. It will also predict the expected underlying true prevalence of *P. falciparum* as a percentage of the total population of the chosen subnational area. When the server completes the model run, the results are plotted almost instantaneously.

The modelled package of interventions comprises the following three aims:

1. Ensure universal access to malaria prevention, diagnosis and treatment.
  - 90% coverage of LLIN modelled as a reduced force of infection dependent on the coverage and the efficacy of LLIN in the GMS setting.
  - 70% of all clinical cases receive early diagnosis and adequate treatment (EDAT) with ACT, modelled as the diversion of the flow of newly infected cases from entering into their respective infection states to the treated state.
2. Accelerate efforts towards elimination and attainment of malaria-free status.
  - Three monthly rounds of MDA targeted on higher prevalence villages. The model assumes that given the linkage between villages due to population mobility, this would translate to an effective coverage of the at-risk population of 50%.
  - The addition of mass vaccination using the RTS,S vaccine to the same population which received the MDA, with three monthly doses of the vaccine delivered simultaneously with the MDA rounds. A booster dose of the vaccine is added after 1 year. Vaccinated individuals are modelled as being partially protected against infection for a period of time before regaining full susceptibility to infection.
3. Transform malaria surveillance into a core intervention.
  - To prevent re-importation of malaria following elimination, the incoming population is modelled as undergoing screening upon entry using HS RDT, and treated if testing positive. This is manifested as a reduction in the rates of importation of infection, depending on the coverage of this intervention and the sensitivity of the test used to each type of infection.

The source code for the modelling tool can be found here [35].

### Interpretation of the results of the modelling tool

**Fig. 5:**
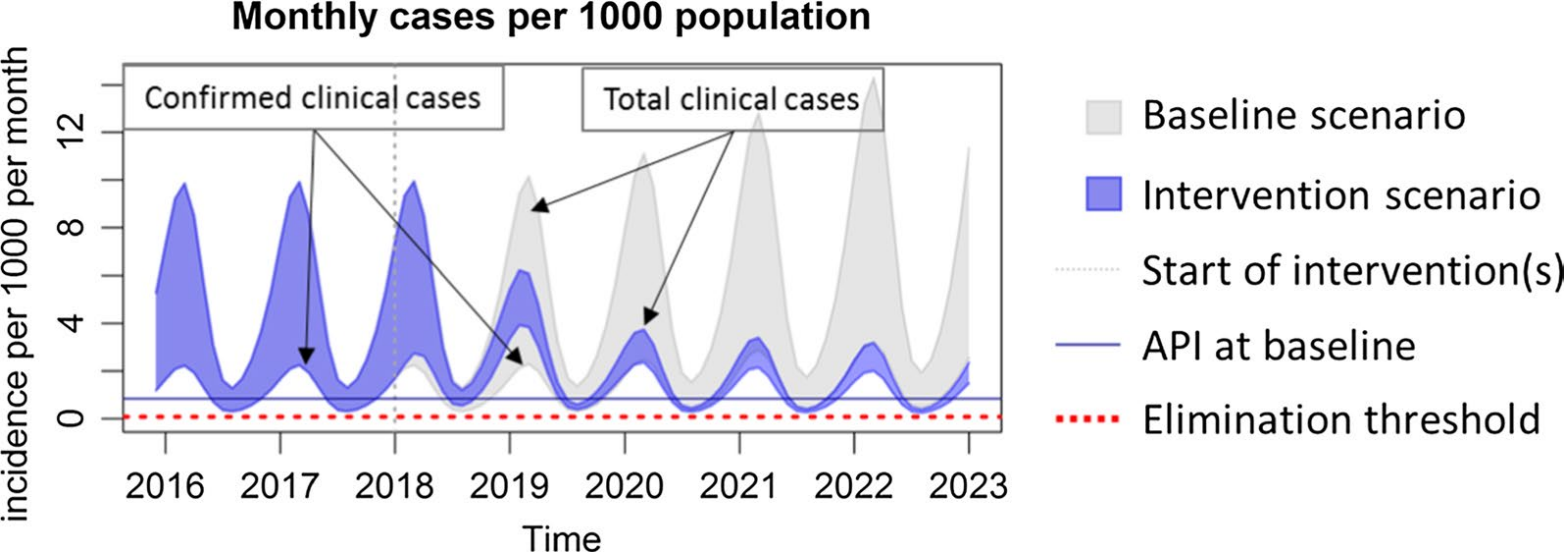
Interpretation of the monthly incidence of malaria from the output of the modelling tool. The incidence is indicated as a range, in which the lower border indicates the estimated incidence of *P. falciparum* detected by passive surveillance, and the upper border the estimated incidence of all infections. Interruption of transmission is achieved when the incidence no longer reaches the elimination threshold

The output of the modelling tool has two graphs. The first one (Fig. 5) plots the model’s prediction for the monthly incidence of clinical malaria cases per 1000 population, making a comparison between the baseline scenario, which is represented by grey lines, and the intervention scenario, which is represented by blue lines. The number of confirmed clinical cases is represented by the lower boundary of the oscillating line, and the total number of clinical cases, inclusive of the clinical cases which are not detected/diagnosed, is represented by the upper boundary of the oscillating line. The number of confirmed cases will be less than the true clinical burden since the model assumes that not all clinical cases are detected and treated by the health system. This is the reason why, under the baseline scenario, the distance between the upper and lower boundary is large. The more cases that are detected by the health system, the more the confirmed cases will reflect the model’s prediction of the true clinical burden. Thus, the range between the upper and lower boundary is narrowed when EDAT coverage is increased.

**Fig. 6:**
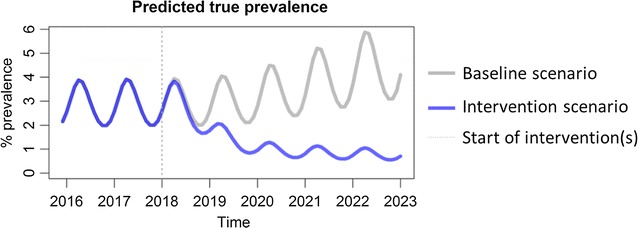
Interpretation of the prevalence of malaria from the output of the modelling tool

The second output of the modelling tool (Fig. 6) shows the model’s prediction for true prevalence. This is defined as the percentage of the population that has a clinical infection (I_C_), a microscopically-detectable asymptomatic infection (I_A_), or a microscopically-undetectable asymptomatic infection (I_U_). It is intended to represent the entire transmission reservoir associated with each scenario.

### Setting up the model baseline and intervention scenarios for Savannakhet

First, a baseline scenario suitable for Savannakhet was set up, the parameters of which can be found in Table 1. According to the Centre for Malariology, Parasitology and Entomology, Lao PDR, this reflects an Annual Parasite Incidence (API) of about 10 detected clinical cases of malaria per 1000 population per year [personal communication], and a mean parasite prevalence of 3.5% [25]. It was assumed that the baseline LLIN coverage was relatively high (70%), while the baseline access to treatment for clinical cases was assumed to be relatively low (25%). Then, the baseline scenario was sequentially augmented with a series of components of a simulated integrated *P. falciparum* elimination package. The coverage of LLIN was increased, resulting in a reduction in the force of infection in those covered by 30% [14]. Early detection and treatment was increased. MDA reached a coverage of 80–95% of a sub-population within three months of each year [9]. The sub-population was assumed to represent 50% of the infectious reservoir. Vaccination using the RTS,S vaccine [36] was included, assuming a peak protective effect of 92% for those who received all three doses of the vaccine, with the protective effect waning to 50% over the following 200 days [11]. The screening of 90% of individuals entering Savannakhet with a high sensitivity RDT was modelled as a reduction in the importation of infected individuals proportional to the assumed sensitivity of the test to each type of infection.

**Table 1:** Assumptions for the baseline scenario in Savannakhet

Parameter	Value	Unit
Baseline API	10	Per 1000 per year
Number of mosquito bites per human per night (peak season)	20	Per night per human
% of all infections that are caught outside the village (forest)	30	%
Baseline % of all clinical cases treated	25	%
Baseline coverage of LLIN	70	%
% of infections averted due to ownership of LLIN	30	%
Baseline coverage of IRS	0	%
% reduction in biting rate due to IRS	15	%
Imported clinical cases	1	Per 1000 per year
Imported asymptomatic microscopically detectable carriers	1	Per 1000 per year
Imported asymptomatic microscopically undetectable carriers	1	Per 1000 per year
% of cases failing treatment in 2018 and before	5	%
% of cases failing treatment in 2019	15	%
% of cases failing treatment in 2020 and after	30	%

These parameters are set up in the “Baseline” tab in the modelling tool

The assumptions on which the model is based are provided in Tables 1, 2, 3, 4 and 5. Elimination is defined as the reduction of detected clinical cases to below the elimination threshold of one case per 1000 population per year. The detailed model structure, equations, and its remaining parameterisation based on [20, 37–45] can be found in Additional file 1.

**Table 2:** Assumptions for the increasing coverage of LLIN and EDAT (universal coverage scenario)

Parameter	Value	Unit
Years to scale up EDAT	1	Year
New % of all clinical cases treated	70	%
Years to universal access to LLIN	1	Year
New bed net use of LLIN	90	%

These parameters are from the “Interventions currently available” tab in the modelling tool

**Table 3:** Assumptions for the focal MDA in addition to universal coverage scenario in Table 2

Parameter	Value	Unit
Effective population coverage (entire province) of focal MVDA in round 1	50	%
Effective population coverage (entire province) of focal MVDA in round 2	50	%
Effective population coverage of focal MVDA in round 3	50	%
Timing of 1st round	September 2018	
Timing of 2nd round	October 2018	
Timing of 3rd round	November 2018	
Months to complete each round	6	Months
Days prophylaxis provided by the ACT	30	Days

These parameters are from the “Interventions under trial: Focal MVDA (hotspot)” tab in the modelling tool

**Table 4:** Assumptions for the MVDA in addition to those in Tables 2 and 3

Parameter	Value	Unit
% protective efficacy of RTS,S with 1st dose	75	%
% protective efficacy of RTS,S with 2nd dose	80	%
% protective efficacy of RTS,S with 3rd dose	92	%
Half-life of vaccine protection	90	days

These parameters are from the “Interventions under trial: Focal MVDA (hotspot)” tab in the modelling tool

**Table 5:** Assumptions for the mass screening and treatment of imported cases in addition to Tables 2, 3 and 4

Parameter	Value	Unit
Years to scale up MSAT	1	Years
New coverage of MSAT	90	%
Sensitivity HS RDT (clinical infections)	99	%
Sensitivity HS RDT (asymptomatic, microscopically detectable)	87	%
Sensitivity HS RDT (asymptomatic, microscopically undetectable)	44	%

These parameters are from the “Interventions under trial: Focal MSAT (mobile)” tab in the modelling tool

## Results

**Fig. 7:**
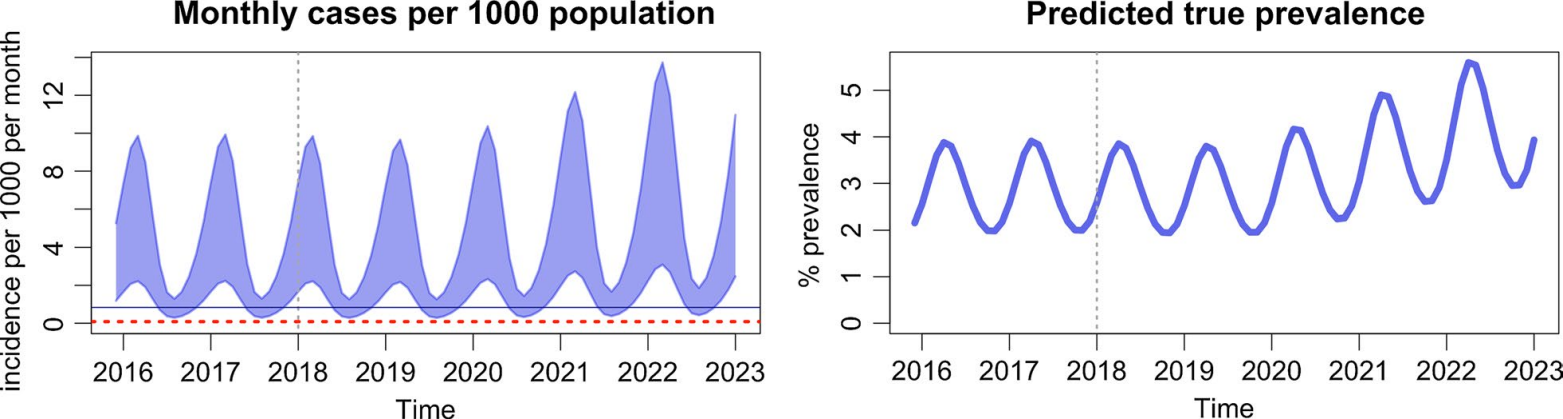
The baseline estimates illustrate the seasonal variability in the presence of spreading anti-malarial resistance

**Fig. 8:**
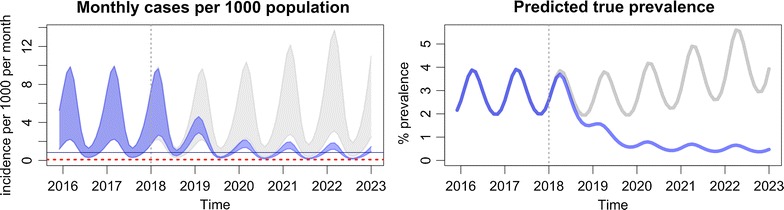
Increasing the coverage of community health workers who distribute LLIN and provide EDAT

The passively detected falciparum malaria incidence in villages in Savannakhet, Lao PDR is estimated on average to be 10 cases per 1000 population per year and, assuming the parameter values in Table 1, the *P. falciparum* infection prevalence is around 3.5% (Fig. 7). VHV are established and distribute LLIN to ensure complete coverage, as well as providing early, accurate diagnosis of malaria and appropriate treatment (Fig. 8, Table 2). This intervention is predicted to slowly reduce the incidence of falciparum infections, but not to elimination levels due to the constant influx of malaria infection imported from outside Savannakhet.

**Fig. 9:**
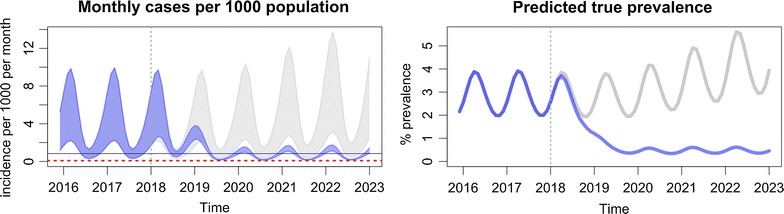
Addition of mass drug administrations (MDA)

**Fig. 10:**
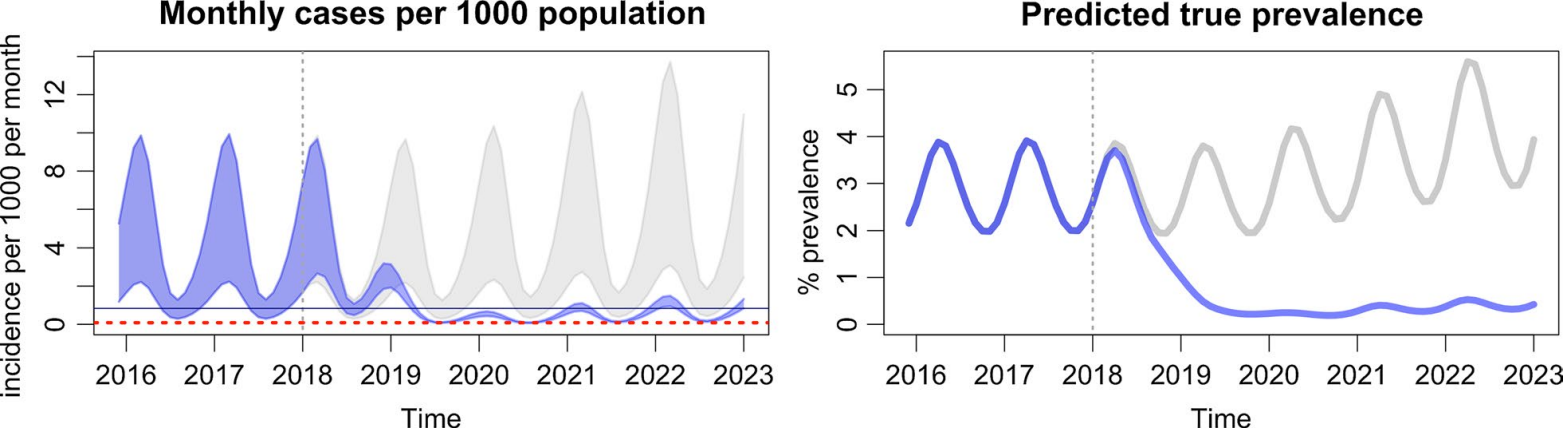
Addition of vaccinations to MDA (MVDA)

The addition of MDA is predicted to accelerate the reduction in both prevalence and incidence in a matter of years (Fig. 9, Table 3), to the levels predicted to be achievable with universal EDAT and LLIN. Re-importation of infections may result in the return to pre-MDA incidence levels in the absence of aggressive case management by VHV. Adding mass vaccination is predicted to interrupt transmission (Fig. 10, Table 4). The added benefit of adding mass vaccination to MDA is further explored in Additional file 1: Figs. S4 and S5. Clinical malaria would then be predicted to drop below the elimination threshold, after which the re-importation of cases would rekindle transmission, resulting in a slow but steady increase in incidence and prevalence.

**Fig. 11:**
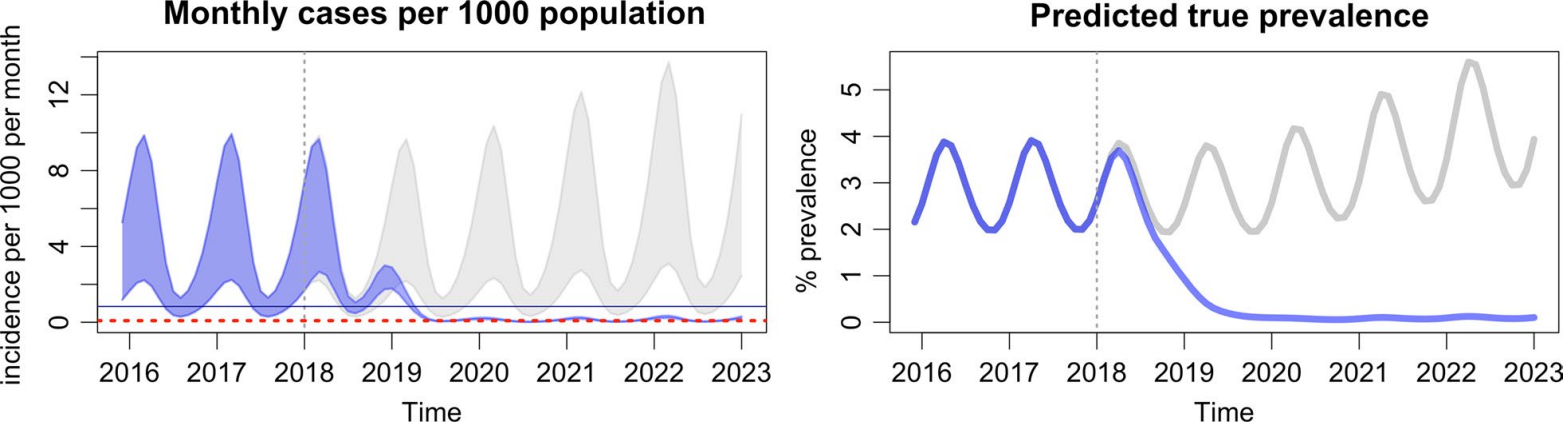
Stopping the importation of infection through screening and treatment of visitors and immigrants

The remaining transmission is driven by imported cases, necessitating the immediate detection and treatment of clinical and subclinical infections entering villages. The acceptance of presumptive anti-malarial therapy is likely to wane with the steady disappearance of clinical malaria. To prevent the return of infections, new arrivals will be screened with HS RDT. Any person found to be infected would be treated with a full course of appropriate anti-malarials. This final step is predicted to prevent reintroduction of infections, and in the model is critical for the permanent interruption of transmission (Fig. 11, Table 5).

## Discussion

The model shows that a sequence of interventions, namely universal access to early diagnosis and adequate treatment, improved access to insecticide-impregnated bed nets, three monthly rounds of MDA together with RTS,S vaccination followed by a booster dose of vaccine, and screening and treatment of imported cases, could eliminate falciparum malaria from Savannakhet over a period of 3 years. Each of these interventions is essential; skipping vaccinations and following MDA with screening and treating newcomers, for example, is insufficient to interrupt transmission.

The findings from this study suggest a need for a multiphasic approach to malaria elimination, in which each phase builds on the achievements of the earlier phase. VHV are a critical foundation for this elimination [46, 47]. Not only do they manage incident malaria cases, their remit also includes the distribution of LLIN to ensure that every resident has access to one, and the timely reporting of malaria cases to the control programme. The additional phases of the malaria elimination programme depend on the services provided by VHV, along with the community engagement efforts developed by VHV together with other malaria project team members. The MDA and vaccination campaigns can only succeed if a large majority of residents participate. Engaging the community in such activities requires trust, especially in the absence of a complete understanding of the complexities of malaria elimination. Only if a VHV has such trust is it be likely that most villagers would participate in MDA and any subsequent mass vaccination campaigns [48]. The model showed that the clearance of malaria would last for just one season in the face of constant reintroduction of infections. Only a rigorous screening programme of newcomers can prevent the resurgence of infections after the initial clearing. As mentioned earlier, it seems unlikely that most newcomers would volunteer to presumptive therapy without a positive diagnostic test. The recent development of HS RDT [12, 20] could therefore be a key element in preventing the reintroduction of infections.

The stacking of sequential interventions requires considerable human and financial resources. However, the combination of interventions suggested in this study can accelerate malaria elimination, particularly in the face of the threat presented by the diminishing efficacy of the tools required for a slow but steady elimination. The early treatment of malaria cases will become increasingly challenging with the spread of multi-drug resistant *P. falciparum* strains. LLIN may also lose their protective efficacy due to the spread of insecticide resistant malaria vectors.

This model was set up to estimate the effect of a variety of combinations of interventions on *P. falciparum* transmission. The elimination of *P. vivax* is equally desirable, but complicated by hypnozoites, the liver stages of *P. vivax*, which can cause a relapse in the absence of a radical cure with 8-aminoquinolines. It is generally assumed that an RTS,S-like vaccine does not provide protection against *P. vivax* infections. The appropriate treatment of all clinical vivax malaria episodes with an 8-aminoquinoline will ultimately result in the elimination of *P. vivax.* The timeframe to eliminate *P. vivax* infection will be longer than that for *P. falciparum*.

## Conclusions

This study found that a sequential implementation of five interventions would be needed to eliminate *P. falciparum* infections from Savannakhet. Such an undertaking carries considerable operational challenges, and has not yet been implemented elsewhere. Preparatory studies to allow for the evaluation of such a strategy of stacking five interventions, including vaccination, are currently underway in Savannakhet.

## Additional file

**Additional file 1.** Model structure and equations.

## Abbreviations

ACT: artemisinin-based combination therapy
API: annual parasite incidence
EDAT: early diagnosis and adequate treatment
G6PD: glucose-6-phosphate dehydrogenase
GMS: Greater-Mekong Subregion
HS RDT: highly sensitive rapid diagnostic test
Lao PDR: Lao People’s Democratic Republic
LLIN: long-lasting insecticidal nets
MDA: mass drug administration
MSAT: mass screening and treatment of imported cases
MVDA: Mass Vaccination and Drug Administration
RDT: rapid diagnostic test
uPCR: ultrasensitive quantitative PCR (polymerase chain reaction)
VHV: village health volunteers
WHO: World Health Organization

## References

[1] WHO (2016). World malaria report 2016.

[2] Centre of Malariology Parasitology and Entomology (2016). National strategic plan for malaria control and elimination 2016–2020.

[3] Imwong M, Suwannasin K, Kunasol C, Sutawong K, Mayxay M, Rekol H (2017). The spread of artemisinin-resistant *Plasmodium falciparum* in the Greater Mekong Subregion: a molecular epidemiology observational study. Lancet Infect Dis..

[4] Ashley EA, Dhorda M, Fairhurst RM, Amaratunga C, Lim P, Suon S (2014). Spread of artemisinin resistance in *Plasmodium falciparum* malaria. N Engl J Med.

[5] Verdrager J (1986). Epidemiology of the emergence and spread of drug-resistant *falciparum* malaria in South-East Asia and Australasia. J Trop Med Hyg..

[6] WHO (2015). Strategy for malaria elimination in the Greater Mekong Subregion (2015–2030).

[7] Mayxay M, Khanthavong M, Chanthongthip O, Imwong M, Pongvongsa T, Hongvanthong B (2012). Efficacy of artemether-lumefantrine, the nationally-recommended artemisinin combination for the treatment of uncomplicated falciparum malaria, in southern Laos. Malar J..

[8] Kaneko A, Taleo G, Kalkoa M, Yamar S, Kobayakawa T, Bjorkman A (2000). Malaria eradication on islands. Lancet.

[9] Newby G, Hwang J, Koita K, Chen I, Greenwood B, von Seidlein L (2015). Review of mass drug administration for malaria and its operational challenges. Am J Trop Med Hyg.

[10] Poirot E, Skarbinski J, Sinclair D, Kachur SP, Slutsker L, Hwang J (2013). Mass drug administration for malaria. Cochrane Database Syst Rev.

[11] Neafsey DE, Juraska M, Bedford T, Benkeser D, Valim C, Griggs A (2015). Genetic diversity and protective efficacy of the RTS,S/AS01 malaria vaccine. N Engl J Med.

[12] Das S, Jang IK, Barney B, Peck R, Rek JC, Arinaitwe E (2017). Performance of a high-sensitivity rapid diagnostic test for *Plasmodium falciparum* malaria in asymptomatic individuals from Uganda and Myanmar and naive human challenge infections. Am J Trop Med Hyg.

[13] We can eradicate malaria—within a generation. https://www.gatesnotes.com/Health/Eradicating-Malaria-in-a-Generation. Accessed 20 Mar 2017.

[14] Sochantha T, Hewitt S, Nguon C, Okell L, Alexander N, Yeung S (2006). Insecticide-treated bednets for the prevention of *Plasmodium falciparum* malaria in Cambodia: a cluster-randomized trial. Trop Med Int Health..

[15] Lao-Oxford-Mahosot hospital-Wellcome Trust Research Unit (2016). Scientific annual report for 2016.

[16] Lubell Y, Dondorp A, Guerin PJ, Drake T, Meek S, Ashley E (2014). Artemisinin resistance–modelling the potential human and economic costs. Malar J..

[17] Malaria in the Asia-Pacific. Modelling the current and potential impact of artemisinin resistance and its containment. http://aplma.org/resources/Papers. Accessed 20 Mar 2017.

[18] Okell L, Slater H, Ghani A, Pemberton-Rossb P, Smith TA, Chitnis N, et al. Consensus modelling evidence to support the design of mass drug administration programmes. In: Malaria Policy Advisory Committee meeting, 16–18 September 2015, Geneva: Background document for Session 1. World Health Organization; 2015.

[19] Maude RJ, Pontavornpinyo W, Saralamba S, Aguas R, Yeung S, Dondorp AM (2009). The last man standing is the most resistant: eliminating artemisinin-resistant malaria in Cambodia. Malar J..

[20] Slater HC, Ross A, Ouedraogo AL, White LJ, Nguon C, Walker PG (2015). Assessing the impact of next-generation rapid diagnostic tests on *Plasmodium falciparum* malaria elimination strategies. Nature.

[21] White LJ, Maude RJ, Pongtavornpinyo W, Saralamba S, Aguas R, Van Effelterre T (2009). The role of simple mathematical models in malaria elimination strategy design. Malar J..

[22] Maude RJ, Socheat D, Nguon C, Saroth P, Dara P, Li G (2012). Optimising strategies for *Plasmodium falciparum* malaria elimination in Cambodia: primaquine, mass drug administration and artemisinin resistance. PLoS ONE.

[23] Parker DM, Landier J, Thu AM, Lwin KM, Delmas G, Nosten FH (2017). Scale up of a *Plasmodium falciparum* elimination program and surveillance system in Kayin State, Myanmar [version 1; referees: 1 approved]. Wellcome Open Res..

[24] Lao Statistics Bureau (2016). Results of population and housing census 2015.

[25] Phommasone K, Adhikari B, Henriques G, Pongvongsa T, Phongmany P, von Seidlein L (2016). Asymptomatic *Plasmodium* infections in 18 villages of southern Savannakhet Province, Lao PDR (Laos). Malar J..

[26] Adhikari B, Pell C, Phommasone K, Soundala X, Kommarasy P, Pongvongsa T (2017). Elements of effective community engagement: lessons from a targeted malaria elimination study in Lao PDR (Laos). Glob Health Action..

[27] Trung HD, Bortel WV, Sochantha T, Keokenchanh K, Briet OJ, Coosemans M (2005). Behavioural heterogeneity of *Anopheles* species in ecologically different localities in Southeast Asia: a challenge for vector control. Trop Med Int Health..

[28] Pongvongsa T, Ha H, Thanh L, Marchand RP, Nonaka D, Tojo B (2012). Joint malaria surveys lead towards improved cross-border cooperation between Savannakhet province, Laos and Quang Tri province, Vietnam. Malar J..

[29] R Core Team. R: a language and environment for statistical computing. R Foundation for Statistical Computing. 2016. https://www.R-project.org/. Accessed 7 Jan 2017.

[30] Chang W, Cheng J, Allaire JJ, Xie Y, McPherson J. Shiny: web application framework for R. The Comprehensive R Archive Network. 2017. https://CRAN.R-project.org/package=shiny. Accessed 7 Jan 2017.

[31] Soetaert K, Petzoldt T, Setzer RW (2010). Solving differential equations in R: package deSolve. J Stat Softw.

[32] Chan K-S, Ripley B. TSA: Time Series Analysis. The Comprehensive R Archive Network. 2012. https://cran.r-project.org/package=TSA. Accessed 7 Jan 2017.

[33] Eddelbuettel D, Francois R (2011). Rcpp: seamless R and C++ integration. J Stat Softw.

[34] Savannakhet model. https://moru.shinyapps.io/savannakhet/. Accessed 18 Nov 2017.

[35] Savannakhet Model Source Code. https://github.com/MAEMOD-MORU/lmrm. Accessed 18 Nov 2017.

[36] Penny MA, Verity R, Bever CA, Sauboin C, Galactionova K, Flasche S (2016). Public health impact and cost-effectiveness of the RTS,S/AS01 malaria vaccine: a systematic comparison of predictions from four mathematical models. Lancet.

[37] Griffin JT, Ferguson NM, Ghani AC (2014). Estimates of the changing age-burden of *Plasmodium falciparum* malaria disease in sub-Saharan Africa. Nat Commun..

[38] Wanji S, Tanke T, Atanga SN, Ajonina C, Nicholas T, Fontenille D (2003). *Anopheles* species of the mount Cameroon region: biting habits, feeding behaviour and entomological inoculation rates. Trop Med Int Health..

[39] Life expectancy Data by WHO region. http://apps.who.int/gho/data/view.main.SDG2016LEXv?lang=en. Accessed 14 May 2017.

[40] Tripura R, Peto TJ, Veugen CC, Nguon C, Davoeung C, James N (2017). Submicroscopic *Plasmodium* prevalence in relation to malaria incidence in 20 villages in western Cambodia. Malar J..

[41] Collins WE, Jeffery GM (1999). A retrospective examination of sporozoite- and trophozoite-induced infections with *Plasmodium falciparum*: development of parasitologic and clinical immunity during primary infection. Am J Trop Med Hyg.

[42] Church LW, Le TP, Bryan JP, Gordon DM, Edelman R, Fries L (1997). Clinical manifestations of *Plasmodium falciparum* malaria experimentally induced by mosquito challenge. J Infect Dis.

[43] Eyles DE, Young MD (1951). The duration of untreated or inadequately treated *Plasmodium falciparum* infections in the human host. J Natl Malar Soc..

[44] Adjuik M, Babiker A, Garner P, Olliaro P, Taylor W, White N (2004). Artesunate combinations for treatment of malaria: meta-analysis. Lancet.

[45] Collins WE, Jeffery GM (1999). A retrospective examination of secondary sporozoite- and trophozoite-induced infections with *Plasmodium falciparum*: development of parasitologic and clinical immunity following secondary infection. Am J Trop Med Hyg.

[46] Dondorp AM, Smithuis FM, Woodrow C, Seidlein LV (2017). How to contain artemisinin- and multidrug-resistant *falciparum* malaria. Trends Parasitol.

[47] Landier J, Parker DM, Thu AM, Carrara VI, Lwin KM, Bonnington CA (2016). The role of early detection and treatment in malaria elimination. Malar J..

[48] Adhikari B, James N, Newby G, von Seidlein L, White NJ, Day NP (2016). Community engagement and population coverage in mass anti-malarial administrations: a systematic literature review. Malar J..

