# Supplementary material for: Towards malaria elimination in Savannakhet, Lao PDR: mathematical modelling driven strategy design
Source: Malar J. 2017 Nov 28;16:483. doi: 10.1186/s12936-017-2130-3 (PMC5706414; doi:10.1186/s12936-017-2130-3)
Supplement: Supplementary file 1 — Additional file 1. Model structure and equations. [file 12936_2017_2130_MOESM1_ESM.docx]

# Model structure and equations

$\frac{dS_{i}}{dt} =\mu P_{i}-\mu_{out}S_{i}+\omega R_{i}-\lambda_{i}S_{i}+\omega_{D}S_{Di}+\left( 1-{\kappa_{MDA}}_{i} \right)m_{i}S_{i-1}-m_{i+1}S_{i}$ ( 1 )

$\frac{dI_{C_{i}}}{dt} = \mu_{C}P_{i}-\mu_{out}I_{C_{i}}+p_{S}\left( 1-\tau\right)\lambda_{i}S_{i}+p_{R}\left( 1-\tau\right)\lambda_{i}R_{i}+p_{R}\left( 1-\tau\right)\lambda_{i}I_{U_{i}} +p_{R}\left( 1-\tau\right)\lambda_{i}I_{A_{i}}-\nu_{C}I_{C_{i}}+\left( 1-{\kappa_{MDA}}_{i} \right)m_{i}{I_{C}}_{i-1}-m_{i+1}{I_{C}}_{i}$ ( 2 )

$\frac{d{I_{A}}_{i}}{dt} = \mu_{A}P_{i}-\mu_{out}{I_{A}}_{i}+\left( 1-p_{S} \right)\lambda_{i}S_{i}+\left( 1-p_{R} \right)\lambda_{i}R_{i}+\left( 1-p_{R} \right)\lambda_{i}{I_{U}}_{i}- p_{R}\lambda_{i}{I_{A}}_{i}+\nu_{C}{I_{C}}_{i}-\nu_{A}{I_{A}}_{i}+f\nu_{T}T_{i}+\left( 1-{\kappa_{MDA}}_{i} \right)m_{i}{I_{A}}_{i-1}-m_{i+1}{I_{A}}_{i}$ ( 3 )

$\frac{d{I_{U}}_{i}}{dt} = \mu_{U}P_{i}-\mu_{out}{I_{U}}_{i}-\lambda_{i}{I_{U}}_{i}-\nu_{U}{I_{U}}_{i}+\nu_{A}{I_{A}}_{i}+\left( 1-{\kappa_{MDA}}_{i} \right)m_{i}{I_{U}}_{i-1}-m_{i+1}{I_{U}}_{i}$ ( 4 )

$\frac{dR_{i}}{dt} = -\mu_{out}R_{i}-\omega R_{i}-\lambda_{i}R_{i}+\nu_{U}{I_{U}}_{i} +\omega_{D}{R_{D}}_{i}+\left( 1-{\kappa_{MDA}}_{i} \right)m_{i}R_{i-1}-m_{i+1}R_{i}$ ( 5 )

$\frac{dT_{i}}{dt} = -\mu_{out}T_{i}+p_{S}\tau\lambda_{i}S_{i}+p_{R}\tau\lambda_{i}R_{i}+p_{R}\tau\lambda_{i}{I_{U}}_{i}+p_{R}\tau\lambda_{i}{I_{A}}_{i}-\nu_{T}T_{i}+m_{i}\left( {\kappa_{MDA}}_{i}\left( {I_{C}}_{i-1}+{I_{A}}_{i-1}+{I_{U}}_{i-1} \right)+T_{i-1} \right)-m_{i+1}T_{i}$ ( 6 )

$\frac{d{S_{D}}_{i}}{dt} = -\mu_{out}{S_{D}}_{i}+\omega{R_{D}}_{i}-\omega_{D}{S_{D}}_{i}+m_{i}\left( {\kappa_{MDA}}_{i}S_{i-1}+{S_{D}}_{i-1} \right)-m_{i+1}{S_{D}}_{i}$ ( 7 )

$\frac{d{R_{D}}_{i}}{dt} = -\mu_{out}{R_{D}}_{i}-\omega{R_{D}}_{i}+\left( 1-f \right)\nu_{T}T_{i}-\omega_{D}{R_{D}}_{i}+m_{i}\left( {\kappa_{MDA}}_{i}R_{i-1}+{R_{D}}_{i-1} \right)-m_{i+1}{R_{D}}_{i}$ ( 8 )

Where$i\in\left\{ 0, 1, 2, 3, 4 \right\}; {m_{0},m}_{5}=0;$ $P_{i}=S_{i}+I_{C_{i}}+{I_{A}}_{i}+{I_{U}}_{i}+R_{i}+T_{i}+{S_{D}}_{i}+{R_{D}}_{i} ; P=\sum_{i=0}^{4} P_{i}$ for the state variables

$S$ for Susceptible; $I_{C}$ for Infected, Clinical cases*;* $I_{A}$ for Infected, Asymptomatic, Patent cases; $I_{U}$ for Infected, Asymptomatic, Sub-microscopic cases; *R* for Recovered cases with immunity; *T* for under Treatment cases; $S_{D}$ for Susceptibles, protected by drug; $R_{D}$ for Recovered cases with both immunity and active drug. Parameters, their descriptions, values/ranges and their references can be found in Table A1.

## Force of infection

The force of infection, $\lambda$, is given by the formula:

$\lambda=\left( \beta\left( 1-\left( 1-\eta\right)\zeta_{IRS} \kappa_{IRS} \right)\left( 1-\zeta_{ITN} \kappa_{ITN} \right)\sum_{i=0}^{4} \left( {I_{C}}_{i}+T_{i}+\rho_{A}{I_{A}}_{i}+\rho_{U}{I_{U}}_{i} \right) \right)/P$ ( 9 )

Where $\beta$ is the effective contact rate (see Equation (10) for its formula), $\eta$is the proportion of infected cases caught in the forest, $\zeta_{IRS}$and $\zeta_{ITN}$ are the effectiveness of IRS (indoor residual spraying) and ITN (insecticide treated nets) respectively, $\kappa_{IRS}$ and $\kappa_{ITN}$ are the coverage of IRS and ITN respectively, $\rho_{A}$ is relative infectivity of patent asymptomatic infections compared with clinical infections, $\rho_{U}$ is relative infectivity of sub-patent asymptomatic infections compared with clinical infections.

The effective contact rate, $\beta$, is calculated as:

$\beta=\left( 1+\alpha cos\left( 2\pi\left( t-\phi\right) \right) \right)\frac{b\epsilon_{h}\epsilon_{m}b_{h}}{\left( b_{h}\epsilon_{h}+\delta_{m} \right)\left( \frac{\gamma_{m}}{\gamma_{m}+\delta_{m}} \right)}$ ( 10 )

Where $\alpha$ is the relative amplitude of the seasonality, $t$ is time in years, $\phi$ is the phase angle (or) the time at peak of seasonality, $b$ is per mosquito rate of biting (ie. How many bites one mosquito performs, which is different from $b_{h}$ which is the no. of bites one human receives), $b_{h}$ is per human biting rate (ie. the no. of bites one human receives), $\epsilon_{h}$ is per bite probability of an infectious mosquito infecting a human, $\epsilon_{m}$ is per bite probability of an infectious human infecting a mosquito, $\delta_{m}$ is the death rate of mosquitos, and $\gamma_{m}$ is the rate of becoming infectious from the latent phase for mosquitos.

Treatment failure, $f$, is assumed to be 5% for year 2018 & before, 15% for year 2019 and 30% for year 2020 & beyond.

## Incidence and prevalence

Depending on the probability of non-immune new cases being clinical, $p_{S}$, the force of infection drives individuals in the S compartment to go to either clinical ($I_{C}$) or asymptomatic, patent ($I_{A}$) compartment. In addition, in order to become clinical cases, they must not be detected by the EDAT system, represented by $\tau$. Asymptomatic cases ($I_{A}$ and $I_{U}$) and recovered cases ($R$) can also be re-infected and become clinical cases again with the same force of infection adjusted by the probability of immune cases becoming clinical, $p_{R}$. Therefore,

$True incidence =\lambda\sum_{i=0}^{4} \left( p_{S}S_{i}+p_{R}\left( R_{i}+{I_{U}}_{i}+{I_{A}}_{i} \right) \right)$ ( 11 )

$Incidence detected by the surveillance system=\tau\lambda\sum_{i=0}^{4} \left( p_{S}S_{i}+p_{R}\left( R_{i}+{I_{U}}_{i}+{I_{A}}_{i} \right) \right)$ ( 12 )

$True prevalence=\frac{\sum_{i=0}^{4} \left( {I_{C}}_{i}+{I_{A}}_{i}+{I_{U}}_{i}+T_{i} \right)}{P}$ ( 13 )

## Interventions

**EDAT** (Early Diagnosis and Adequate Treatment) works by whisking away a part of newly infected clinical cases from $S, R, I_{A} and I_{U}$ into the treatment compartment, $T$. It depends on the coverage and effectiveness of EDAT which is represent by $\tau$. Equation (12) also represents the number of cases detected and treated by the EDAT system.

**ITN** (Insecticide Treated Nets) and **IRS** (Indoor-residual Spraying) are modelled as in Equation (9).

EDAT, ITN and IRS are assumed to acquire their coverage gradually, depending on the time it takes to scale up. This is modelled by gradual scaling function:

$g(t)=\left\{ \begin{aligned} 0, t\leq t_{int} \\ \left( \frac{t-t_{int}}{d_{scale}} \right), & t_{int}<t\leq\left( t_{int}+d_{scale} \right) \\ 1, t>(t_{int}+d_{scale}) \end{aligned} \right.$ ( 14 )

Where $t$ is time in year, $t_{int}$ is the time at which the intervention starts, $d_{scale}$ is the duration it takes for the intervention to reach the target coverage. The gradual scaling function can be sketched as figure A1.

**Figure A1: Gradual scaling of coverage**


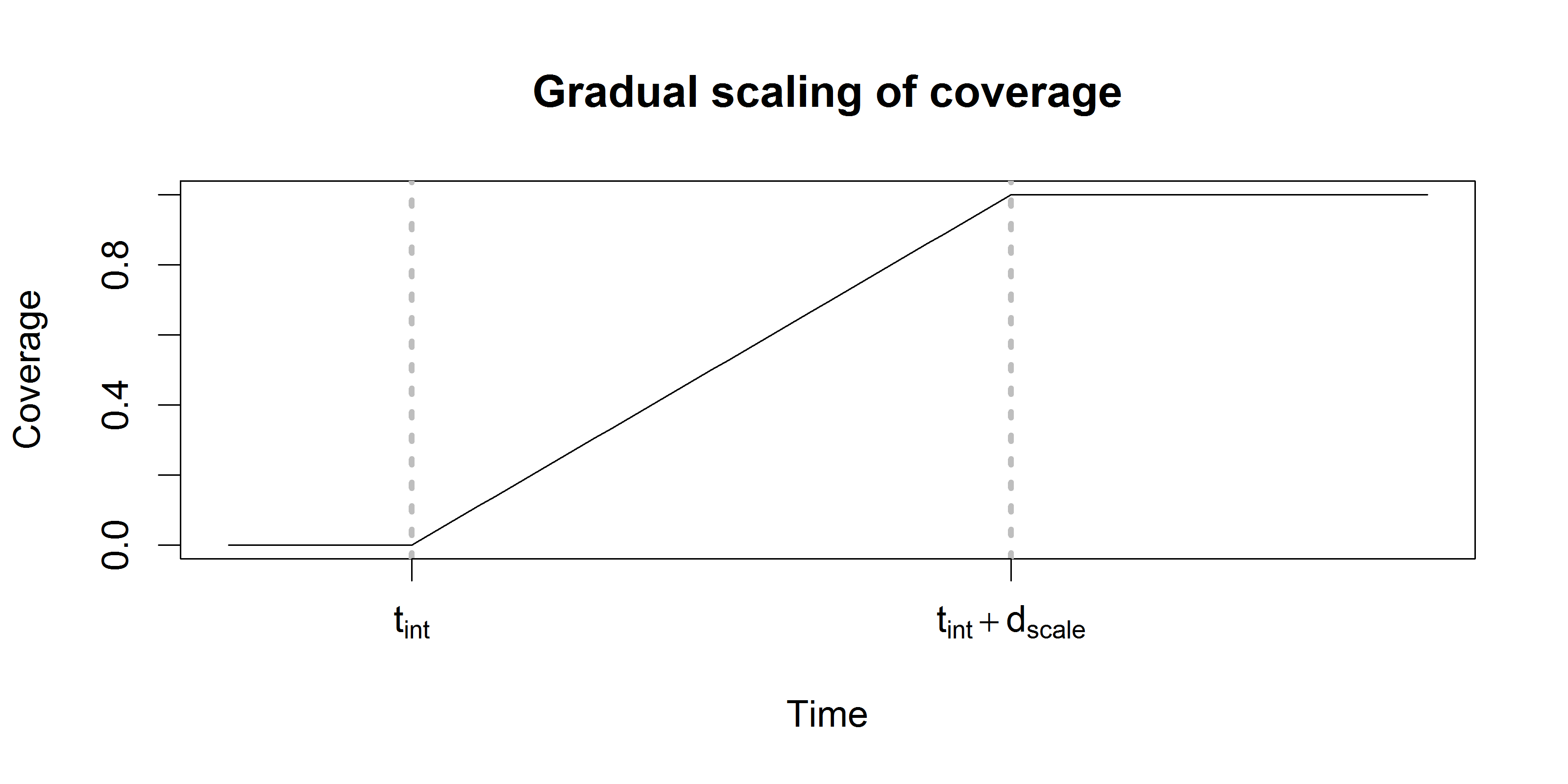


**MDA** (Mass Drug Administration) moves individuals from $S_{i} and R_{i}$ to their respective counterpart on the next layer where drug protection is present, $S_{D_{i+1}}and R_{D_{i+1}}$, and from ${I_{A}}_{i}, {I_{C}}_{i}, {I_{U}}_{i}$ to $T_{i+1}.$ The rate of MDA can be seen in Equations (1-8), represented by $m_{i}$, which is as follows:

$m_{i}(t)=\left\{ \begin{aligned} 0, &t\leq{t_{MDA}}_{i}\vee t>\left( {t_{MDA}}_{i}+d_{MDA} \right) \\ -ln(1-{\kappa_{m}}_{i})/d_{MDA}, &{t_{MDA}}_{i}<t\leq\left( {t_{MDA}}_{i}+d_{MDA} \right) \end{aligned} \right.$ ( 15 )

Where $i\in\left\{ 1, 2, 3 \right\}$. In order to be structurally possible for a booster dose of vaccine, $m_{4}$ is equal to $m_{3}$ while the coverage of MDA corresponding to $m_{4}$ is set to 0. $m_{0}$ and $m_{5}$ are 0, as stated previously. $t$ is time in year, ${t_{MDA}}_{i}$ is the time at which the $i$^th^ MDA starts, $d_{MDA}$ is the duration to complete each MDA round, ${\kappa_{m}}_{i}$ is the local population coverage of the $i$^th^ round of MDA (eg. Coverage of households within a village). The rate of MDA can be sketched as in figure A2.

**Figure A2: Rate of MDA over time**


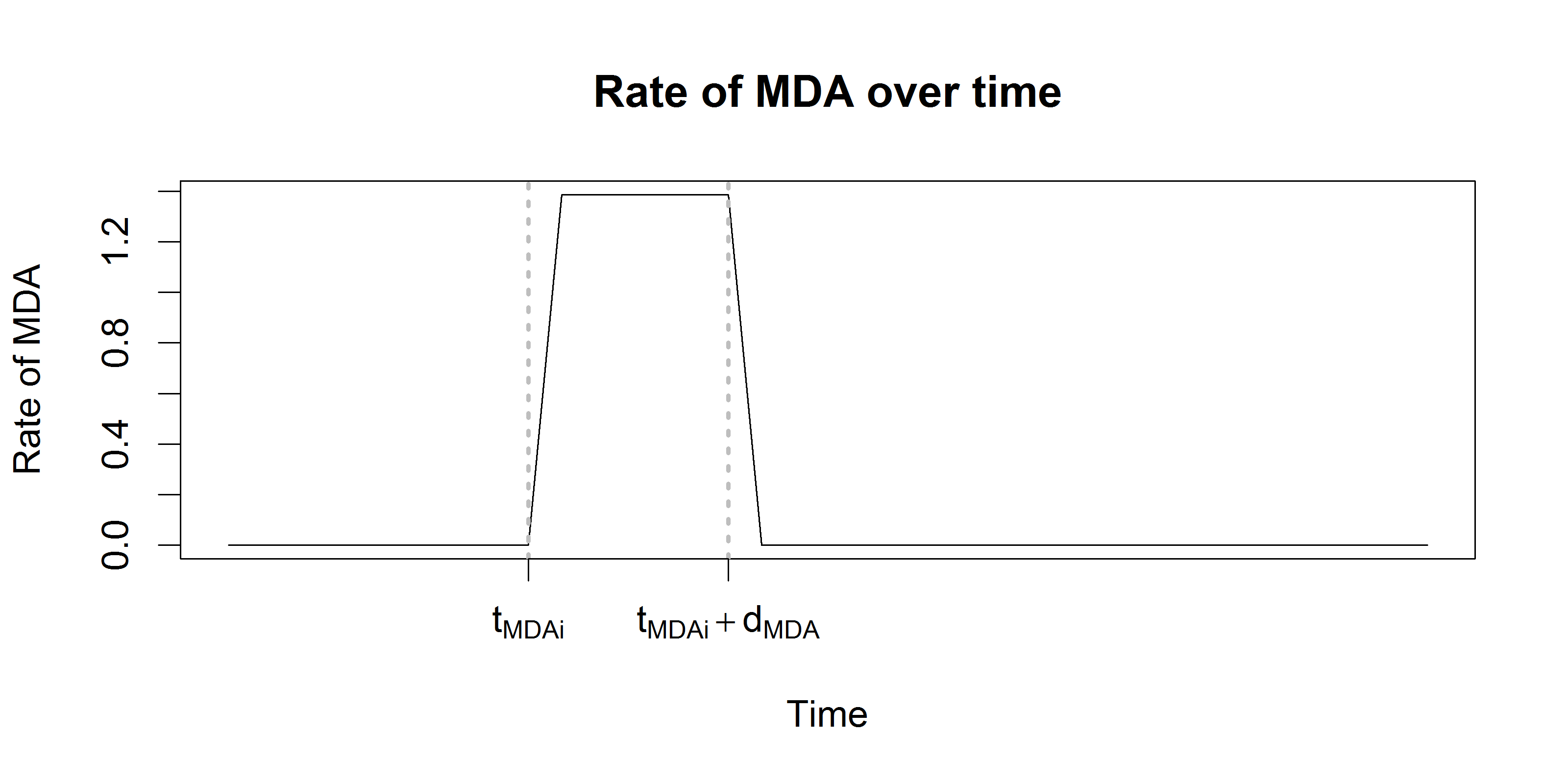


**Vaccination**, which we’re assuming to happen simultaneously with MDA, adjusts $\lambda$ by the parameter, $v_{i}$, so that

$\lambda_{i}=\left( 1-v_{i} \right)\lambda$ ( 16 )

Where $i\in\left\{ 1, 2, 3, 4 \right\}$. $v_{i}$ is also time-dependent. Therefore,

$v_{i}(t)=\left\{ \begin{matrix} 0, & t\leq{t_{MDA}}_{i} \\ {\zeta_{v}}_{i}\left( \frac{v_{h}}{d_{MDA}\ln\left( 2 \right)}+\left( 1-\frac{v_{h}}{d_{MDA}\ln\left( 2 \right)} \right)e^{-\left( t-{t_{MDA}}_{i} \right)\frac{\ln\left( 2 \right)}{v_{h}}} \right), & {t_{MDA}}_{i}<t\leq\left( {t_{MDA}}_{i}+d_{MDA} \right) \\ \zeta_{v_{i}}\left( \frac{v_{h}}{d_{MDA}\ln\left( 2 \right)}+\left( 1-\frac{v_{h}}{d_{MDA}\ln\left( 2 \right)} \right)e^{\frac{-\left( \ln\left( 2 \right) d_{MDA} \right)}{v_{h}}} \right)e^{-\left( t-{t_{MDA}}_{i}-d_{MDA} \right)\frac{\ln\left( 2 \right)}{v_{h}}}, & t>\left( {t_{MDA}}_{i}+d_{MDA} \right) \end{matrix} \right.$ ( 17 )

Effect of vaccine over time can be sketched as figure A3.

**Figure A3: Effect of vaccine over time**

**
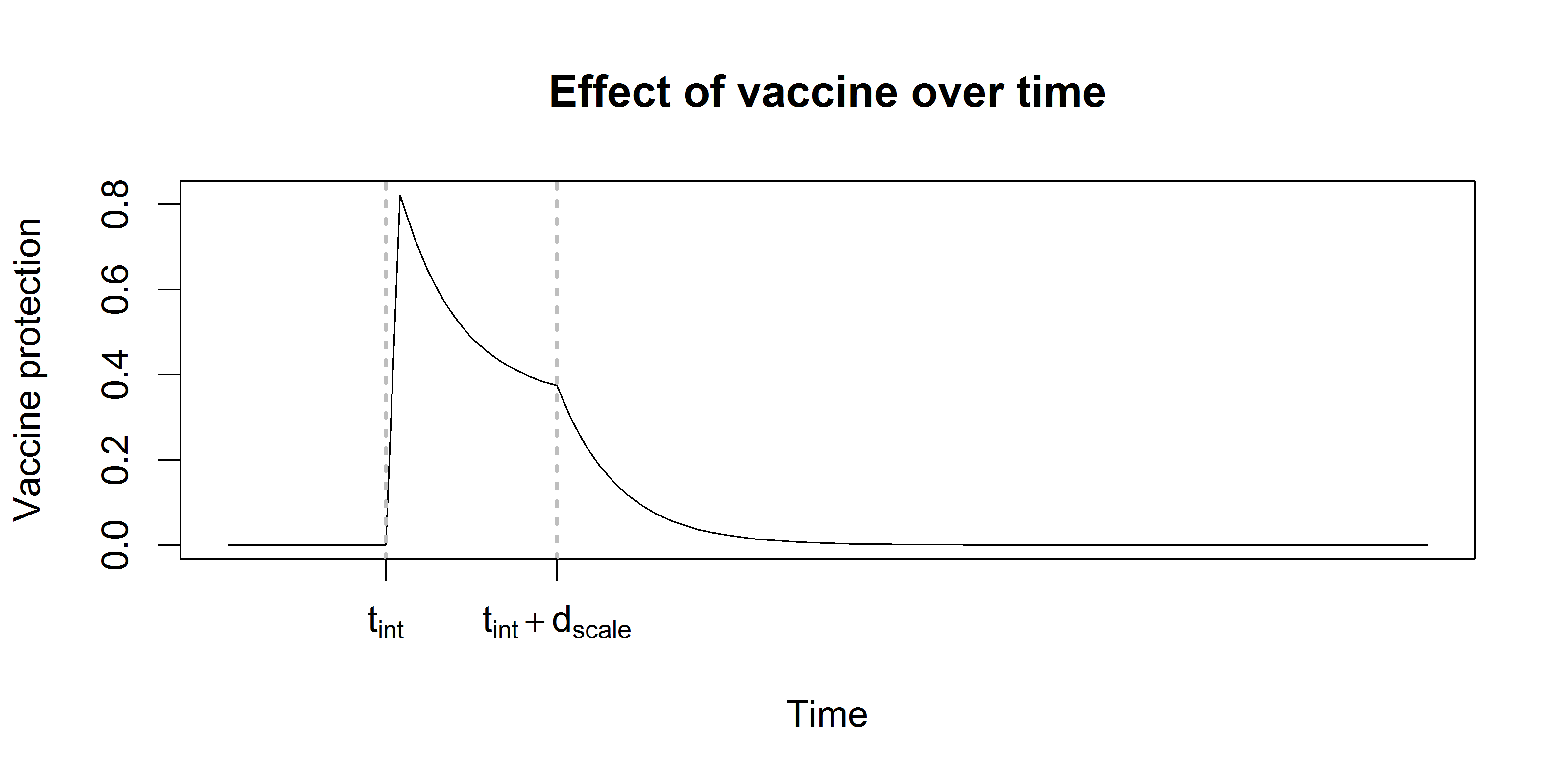
**

**Figure A4: Additional benefit of vaccination on top of EDAT, LLIN, and MDA. The grey baseline here already include EDAT, LLIN, and MDA.**

**
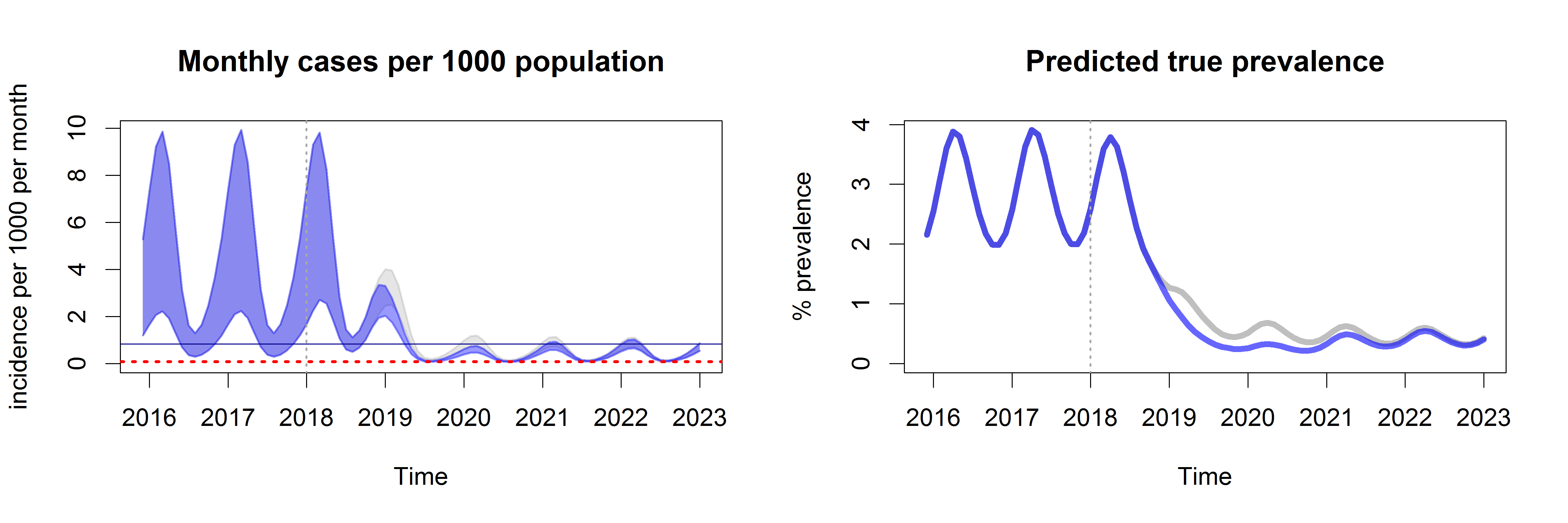
**

**Figure A5: Integrated strategy with EDAT, LLIN, MDA, and MSAT, but without vaccination. The grey baseline here already include EDAT, LLIN, and MDA.**

**
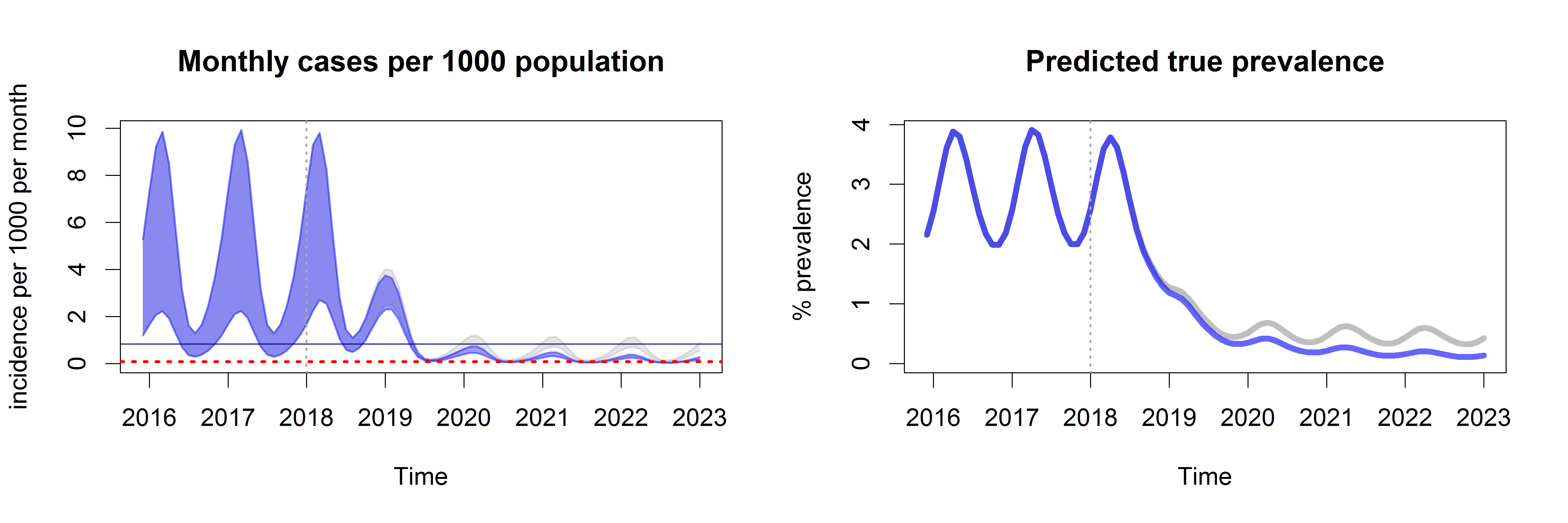
**

**MSAT** (Mass Screen and Treat) affects the importation rates $\mu_{C}, \mu_{A}, and \mu_{U}$ by reducing them depending on the coverage and sensitivity to detect such imported cases. The general formula is below

$\mu_{j_{MSAT}}=\left( 1-\chi_{MSAT}\xi_{j}\kappa_{MSAT} \right)\mu_{j}$ ( 18 )

Where $j\in\left\{ C, A, U \right\}$, and ${\mu_{j}}_{MSAT}$ is the new importation rate after MSAT, $\mu_{j}$ is the original impartation rate, $\chi_{MSAT}$ is the presence or absence of MSAT, $\xi_{j}$ is the sensitivity of detecting $j$, $\kappa_{MSAT}$ is the coverage of MSAT.

# Equations in the source code

The equations in this Additional file are signposted in the source code files “modGMS.cpp” and “app.R” with the corresponding equation numbers. The source code can be found in <https://github.com/MAEMOD-MORU/lmrm>.

# Table A1: Parameters

| **#** | **Name** | **Description** | **Value** | **Unit** | **Reference** | **User Input** | **R script** |
| --- | --- | --- | --- | --- | --- | --- | --- |
| 1 | $\alpha$ | Relative amplitude of the seasonality | 0.7 | - | - | n | alpha |
| 2 | $\beta$ | Effective contact rate | - | - | - | n^*^ | beta |
| 3 | $\gamma_{m}$ | Rate of becoming infectious from the latent phase for mosquitos | 365/10 | /year | [1] | n | gamma_m |
| 4 | $\delta_{m}$ | Death rate of mosquitos | 365/14 | /year | [2, 3] | n | delta_m |
| 5 | $\epsilon_{h}$ | Per bite probability of an infectious mosquito infecting a human | 0.23 | proportion | - | n | epsilon_h |
| 6 | $\epsilon_{m}$ | Per bite probability of an infectious human infecting a mosquito | 0.5 | proportion | - | n | epsilon_m |
| 7 | $\zeta_{IRS}$ | Effectiveness of IRS (indoor residual spraying), reduction in risk provided by IRS | 0.15 | proportion | - | y | effIRS |
| 8 | $\zeta_{ITN}$ | Effectiveness of ITN (insecticide treated nets), proportion of new infections averted due to ownership of ITN | 0.30 | proportion | [4] | y | effITN |
| 9 | ${\zeta_{v}}_{i}$ | Protective efficacy after $i$^th^ doses of RTS,S | Variable | proportion | [5] | y | effv_i |
| 10 | $\eta$ | Proportion of infected cases caught in the forest | 0.30 | proportion | - | y | eta |
| 11 | ${\kappa_{m}}_{1}$ | Population coverage of 1^st^ MDA round in a focal area (e.g., Coverage in a village) | 0.80 | proportion | [6] | n | cm_1 |
| 12 | ${\kappa_{m}}_{2}$ | Proportion of 1^st^ MDA round population who gets the 2^nd^ MDA round | 0.95 | proportion | [6] | n | cm_2 |
| 13 | ${\kappa_{m}}_{3}$ | Proportion of 2^nd^ MDA round population who gets the 3^rd^ MDA round | 0.95 | proportion | [6] | n | cm_3 |
| 14 | $\kappa_{IRS}$ | Coverage of IRS | 0 | proportion | - | y | covIRS |
| 15 | $\kappa_{ITN}$ | Coverage of ITN | 0.70 | proportion | - | y | covITN |
| 16 | ${\kappa_{MDA}}_{i}$ | Effective coverage of focal MDA $i$^th^ round out of the whole location/region under consideration | 0.5 | proportion | - | y | cmda_i |
| 17 | $\kappa_{MSAT}$ | Coverage of MSAT | 0.90 | proportion | - | y | covMSAT |
| 18 | $\lambda$ | Force of infection | - | - | - | n^*^ | lambda |
| 19 | $\mu$ | Birth/death rate | 1/69 | /year | [7] | n | mu |
| 20 | $\mu_{A}$ | Rate of importation of asymptomatic patent cases | 1 | /year/1000 | [8] | y | muA |
| 21 | $\mu_{C}$ | Rate of importation of clinical cases | 1 | /year/1000 | [8] | y | muC |
| 22 | $\mu_{out}$ | Death rate + emigration rates for malaria cases | - | - | - | n^*^ | mu_out |
| 23 | $\mu_{U}$ | Rate of importation of asymptomatic non-patent cases | 1 | /year/1000 | [8] | y | muU |
| 24 | $\nu_{A}$ | Rate of transition from asymptomatic patent state (IA) to asymptomatic non-patent state (IU) | 365/60 | /year | [9] | n | nuA |
| 25 | $\nu_{C}$ | Rate of relief from clinical symptoms in absence of treatment | 365/3 | /year | [10] | n | nuC |
| 26 | $\nu_{U}$ | Rate of transition from asymptomatic non-patent state (IU) to recovered state (R) | 365/100 | /year | [11] | n | nuU |
| 27 | $\nu_{Tr}$ | Recovery rate after treatment ACT | 365/14 | /year | [12] | n | nuTr |
| 28 | $\nu_{Trp}$ | Recovery rate after treatment ACT + primaquine | 365/7 | /year | - | n | nuTrp |
| 29 | $\xi_{A}$ | Sensitivity of the detecting an asymptomatic, patent (microscopically detectable) case with MSAT | 0.87 | proportion | - | y | MSATsensA |
| 30 | $\xi_{C}$ | Sensitivity of the detecting a Clinical case with MSAT | 0.99 | proportion | - | y | MSATsensC |
| 31 | $\xi_{U}$ | Sensitivity of the detecting an asymptomatic, non-patent (microscopically undetectable) case with MSAT | 0.44 | proportion | [13] | y | MSATsensU |
| 32 | $\rho_{A}$ | Relative infectivity of super-microscopic asymptomatic infections compared with clinical infections | 0.55 | proportion | [14] | n | rhoa |
| 33 | $\rho_{U}$ | Relative infectivity of sub-microscopic asymptomatic infections compared with clinical infections | 0.17 | proportion | [14] | n | rhou |
| 34 | $\tau$ | Coverage and effect of early diagnosis and treatment (EDAT) | - | - | - | n^*^ | tau |
| 35 | $\phi$ | Phase angle of seasonality | 0.0 | - | - | n | phi |
| 36 | $\chi_{EDAT}$ | On/off switch for EDAT | 1 or 0 | - | - | y | EDATon |
| 37 | $\chi_{IRS}$ | On/off switch for IRS | 1 or 0 | - | - | y | IRSon |
| 38 | $\chi_{ITN}$ | On/off switch for ITN | 1 or 0 | - | - | y | ITNon |
| 39 | $\chi_{MDA}$ | On/off switch for MDA | 1 or 0 | - | - | y | MDAon |
| 40 | $\chi_{MSAT}$ | On/off switch for Mass Screen and Treat (MSAT) for imported cases | 1 or 0 | - | - | y | MSATon |
| 41 | $\omega$ | Rate of immunity loss | ½ | /year | - | n | omega |
| 42 | $\omega_{D}$ | Rate of loss of protection by drug (ACT) | 365/30 | /year | - | y | lossd |
| 43 | $\omega_{v}$ | half-life of vaccine protection (days) | 90 | Days | [5] | n | vh |
| 44 | b | Per mosquito rate of biting (i.e. the no. of bites one mosquito performs) | 365/3 | /year | - | n | b |
| 45 | $b_{\mathrm{hMax}}$ | Per human biting rate (i.e. the no. of bites one human receives) in the peak season | 20 | /night/human | - | y | bh_max |
| 46 | $d_{MDA}$ | duration to complete each MDA round | 6 | months | - | y | dm |
| 47 | $p_{R}$ | Proportion of all immune new infections that are clinical | 0.20 | proportion | [15] | n | pr |
| 48 | $p_{S}$ | Proportion of all non-immune new infections that are clinical | 0.90 | proportion | [9] | n | ps |
| 49 | ${t_{m}}_{i}$ | Timing of $i$^th^ round of MDA | 09-11/2018 | Time-point | - | y | tm_i |
| 50 | $v_{i}$ | Vaccine effect of $i$^th^ round | - | - | - | n^*^ | v_i |

^*^ Calculated within the model

N.B. Percentages are used in the user interface of the modelling tool to be user friendly and thus, also in the tables in the main manuscript. But, those percentages are transformed into proportions (represented in the table above) before the actual model run.

1. Matuschewski K. Getting infectious: formation and maturation of Plasmodium sporozoites in the Anopheles vector. Cell Microbiol. 2006;8(10):1547-56. doi: 10.1111/j.1462-5822.2006.00778.x. PubMed PMID: 16984410.

2. Beck-Johnson LM, Nelson WA, Paaijmans KP, Read AF, Thomas MB, Bjørnstad ON. The Effect of Temperature on Anopheles Mosquito Population Dynamics and the Potential for Malaria Transmission. PLoS ONE. 2013;8(11):e79276. doi: 10.1371/journal.pone.0079276.

3. Charlwood JD, Smith T, Billingsley PF, Takken W, Lyimo EOK, Meuwissen JHET. Survival and infection probabilities of anthropophagic anophelines from an area of high prevalence of Plasmodium falciparum in humans. Bulletin of Entomological Research. 2009;87(5):445-53. Epub 07/01. doi: 10.1017/S0007485300041304.

4. Sochantha T, Hewitt S, Nguon C, Okell L, Alexander N, Yeung S, et al. Insecticide-treated bednets for the prevention of Plasmodium falciparum malaria in Cambodia: a cluster-randomized trial. Tropical medicine & international health : TM & IH. 2006;11(8):1166-77. Epub 2006/08/15. doi: 10.1111/j.1365-3156.2006.01673.x. PubMed PMID: 16903880.

5. Neafsey DE, Juraska M, Bedford T, Benkeser D, Valim C, Griggs A, et al. Genetic Diversity and Protective Efficacy of the RTS,S/AS01 Malaria Vaccine. N Engl J Med. 2015;373(21):2025-37. Epub 2015/10/22. doi: 10.1056/NEJMoa1505819. PubMed PMID: 26488565; PubMed Central PMCID: PMCPMC4762279.

6. Newby G, Hwang J, Koita K, Chen I, Greenwood B, von Seidlein L, et al. Review of mass drug administration for malaria and its operational challenges. Am J Trop Med Hyg. 2015;93(1):125-34. Epub 2015/05/28. doi: 10.4269/ajtmh.14-0254. PubMed PMID: 26013371; PubMed Central PMCID: PMCPMC4497884.

7. WHO. Life expectancy Data by WHO region [01/03/2017]. Available from: <http://apps.who.int/gho/data/view.main.SDG2016LEXv?lang=en>.

8. Tripura R, Peto TJ, Veugen CC, Nguon C, Davoeung C, James N, et al. Submicroscopic Plasmodium prevalence in relation to malaria incidence in 20 villages in western Cambodia. Malaria Journal. 2017;16(1):56. doi: 10.1186/s12936-017-1703-5.

9. Collins WE, Jeffery GM. A retrospective examination of sporozoite- and trophozoite-induced infections with Plasmodium falciparum: development of parasitologic and clinical immunity during primary infection. Am J Trop Med Hyg. 1999;61(1 Suppl):4-19. Epub 1999/08/04. PubMed PMID: 10432041.

10. Church LW, Le TP, Bryan JP, Gordon DM, Edelman R, Fries L, et al. Clinical manifestations of Plasmodium falciparum malaria experimentally induced by mosquito challenge. J Infect Dis. 1997;175(4):915-20. PubMed PMID: 9086149.

11. Eyles DE, Young MD. The duration of untreated or inadequately treated Plasmodium falciparum infections in the human host. J Natl Malar Soc. 1951;10(4):327-36. PubMed PMID: 14908561.

12. Adjuik M, Babiker A, Garner P, Olliaro P, Taylor W, White N, et al. Artesunate combinations for treatment of malaria: meta-analysis. Lancet. 2004;363(9402):9-17. PubMed PMID: 14723987.

13. Das S, Jang IK, Barney B, Peck R, Rek JC, Arinaitwe E, et al. Performance of a High-Sensitivity Rapid Diagnostic Test for Plasmodium falciparum Malaria in Asymptomatic Individuals from Uganda and Myanmar and Naive Human Challenge Infections. Am J Trop Med Hyg. 2017. Epub 2017/08/19. doi: 10.4269/ajtmh.17-0245. PubMed PMID: 28820709.

14. Slater HC, Ross A, Ouedraogo AL, White LJ, Nguon C, Walker PG, et al. Assessing the impact of next-generation rapid diagnostic tests on Plasmodium falciparum malaria elimination strategies. Nature. 2015;528(7580):S94-101. Epub 2015/12/04. doi: 10.1038/nature16040. PubMed PMID: 26633771.

15. Collins WE, Jeffery GM. A retrospective examination of secondary sporozoite- and trophozoite-induced infections with Plasmodium falciparum: development of parasitologic and clinical immunity following secondary infection. Am J Trop Med Hyg. 1999;61(1 Suppl):20-35. Epub 1999/08/04. PubMed PMID: 10432042.
